# Towards the Development of Voice Assistants for Social Interaction in People Living with Alzheimer's Disease

**DOI:** 10.1002/alz70858_104569

**Published:** 2025-12-26

**Authors:** Lili Marlene Camacho‐Bustamante, Sergio Alberto Navarro‐Tuch, Alberto Agustín Palacios‐García, Rogelio Bustamante‐Bello

**Affiliations:** ^1^ Tecnologico de Monterrey, Mexico City, VL, Mexico; ^2^ Tecnologico de Monterrey, Mexico City, DF, Mexico

## Abstract

**Background:**

Social interaction plays a critical role in the progression of psychobehavioral symptoms in individuals with Alzheimer's disease, significantly enhancing their resilience against the progressive effects of the disease. Numerous studies have highlighted that an active social support network not only alleviates the emotional impact of Alzheimer's disease but also improves persons’ quality of life by fostering a sense of belonging and reducing feelings of isolation. However, many individuals with Alzheimer's disease face challenges in maintaining social interactions due to barriers associated with cognitive changes, communication impairments, or living in solitude. These limitations, coupled with societal stigma, can exacerbate their isolation and negatively impact their well‐being.

**Method:**

A comprehensive analysis was conducted to identify the key factors in designing voice assistants for individuals with Alzheimer's disease. The analysis focused on three primary aspects: the specific symptoms of Alzheimer's, the emotional and social well‐being of individuals, and the typical age at which the disease develops. Factors such as cognitive decline, memory impairment, and communication difficulties were considered to ensure the technology's usability across different stages of the disease.

**Result:**

This article explores the key requirements for designing voice assistants tailored to individuals with Alzheimer's disease from an integral perspective. The proposed framework emphasizes the consideration of factors such as age, symptom progression, and user needs, ensuring that the design is accessible, adaptable, and usable. By addressing these aspects holistically, the framework offers a comprehensive approach for adapting and designing voice assistants that specifically meet the needs of individuals with Alzheimer's disease.

**Conclusion:**

The proposed framework for designing voice assistants offers a comprehensive approach to improving the quality of life of individuals with Alzheimer's disease by focusing on social interaction, ensuring that these tools are effectively tailored to their needs.

